# The Role of Specific Components of a Plant-Based Diet in Management of Dyslipidemia and the Impact on Cardiovascular Risk

**DOI:** 10.3390/nu12092671

**Published:** 2020-09-01

**Authors:** Elke A. Trautwein, Sue McKay

**Affiliations:** 1 Trautwein Consulting, 58097 Hagen, Germany; 2 Upfield Research & Development, 3071 JL Rotterdam, The Netherlands; Sue.Mckay@Upfield.com

**Keywords:** review, CVD, dyslipidemia, cardiovascular health, dietary fats, dietary fiber, phytosterols, plant-based diet, dietary pattern, sustainability

## Abstract

Convincing evidence supports the intake of specific food components, food groups, or whole dietary patterns to positively influence dyslipidemia and to lower risk of cardiovascular diseases (CVD). Specific macro- and micro-components of a predominantly plant-based dietary pattern are vegetable fats, dietary fibers, and phytonutrients such as phytosterols. This review summarizes the current knowledge regarding effects of these components on lowering blood lipids, i.e., low-density lipoprotein cholesterol (LDL-C) and on reducing CVD risk. The beneficial role of a plant-based diet on cardiovascular (CV) health has increasingly been recognized. Plant-based dietary patterns include a Mediterranean and Nordic diet pattern, the dietary approaches to stop hypertension (DASH), and Portfolio diet, as well as vegetarian- or vegan-type diet patterns. These diets have all been found to lower CVD-related risk factors like blood LDL-C, and observational study evidence supports their role in lowering CVD risk. These diet patterns are not only beneficial for dyslipidemia management and prevention of CVD but further contribute to reducing the impact of food choices on environmental degradation. Hence, the CV health benefits of a predominantly plant-based diet as a healthy and environmentally sustainable eating pattern are today recommended by many food-based dietary as well as clinical practice guidelines.

## 1. Introduction

Cardiovascular diseases (CVD), which include coronary heart disease (CHD), cerebrovascular disease (stroke), and peripheral vascular disease, are the most common non-communicable diseases globally, accounting for an estimated 31% of all deaths worldwide [1]. The underlying cause of CVD is atherosclerosis which is a progressive (and reversible) disease of the blood vessels. By managing risk factors associated with atherosclerosis it is possible to slow down (or reverse) the development of the disease and ultimately reduce the risk of CVD [2]. Underlying risk factors include elevated blood pressure, dyslipidemia, overweight/obesity, and type 2 diabetes mellitus (T2DM), as well as behavioral risk factors such as smoking, unhealthy diet, and physical inactivity [2]. Dyslipidemia is a metabolic abnormality leading to an increase in circulating concentrations of blood cholesterol (C) and triglycerides (TG). It is characterized by elevated low-density lipoprotein cholesterol (LDL-C), also known as hypercholesterolemia, and often combined with low concentrations of high-density lipoprotein cholesterol (HDL-C) and elevated TG, mainly as TG-rich lipoproteins (TRL) such as chylomicrons and very-low-density lipoprotein (VLDL).

Elevated LDL-C is a causal risk factor for CVD [3] and lowering LDL-C concentrations is the primary target for treatment and prevention of CVD [2]. Elevated TG, and especially TRL, are also treatment targets recommended in guidelines for the management of dyslipidemia [2].

Adopting a healthy diet and lifestyle should always be the cornerstone when seeking to lower LDL-C (and TG) concentrations [2]. Based on the severity of dyslipidemia and the total CVD risk score, additional pharmacological therapy may be recommended. With adequate changes in diet and lifestyle, about 80% of (premature) CVD mortality may be prevented [4].

The role of a healthy dietary pattern in the prevention of CVD has well been recognized [5,6,7]. Evidence supports the intake of specific nutrients, food groups, or certain dietary patterns to positively influence dyslipidemia and promote the prevention of CVD. A healthy dietary pattern is also a major determinant of environmental sustainability. Current food production practices contribute to environmental degradation in several ways, e.g., through greenhouse-gas emissions, fresh-water withdrawal, and land use [8]. Consequently, authorities are starting to adapt their food-based dietary guidelines to reflect a shift towards a predominantly plant-based diet. These recommendations aim to reduce the intake of animal-based foods for both health and environmental reasons [9,10].

Predominantly plant-based dietary patterns emphasize a higher intake of fruits, vegetables, legumes, whole-grain products, nuts, seeds, and vegetable oils and limited intake of foods from animal origin such as low- or non-fat dairy, lean meat, and fish [7]. Plant-based dietary patterns vary from flexitarian (with low consumption of meat) to pescatarian or lacto/ovo-vegetarian to vegan, where plant-based foods represent most or all the food in the diet.

In view of macronutrients, specific for a plant-based diet are the intake of more complex carbohydrates and plant-based proteins next to a lower total fat intake, especially fewer saturated and trans fats and more unsaturated fatty acids. Plant-based diets are also higher in dietary fiber (DF). In addition, they contain modest amount of phytosterols (PS) next to other bioactive plant-derived compounds often called phytonutrients, which are associated with positive health effects. This review focuses on PS because of their proven cholesterol-lowering effect which allows them to carry an authorized health claim. Other phytonutrients, such as polyphenols, lignans, and carotenoids, as well as vitamins like folic acid, are out of scope for this review.

This narrative review, based on evidence from reviews, systematic reviews, meta-analyses, and randomized controlled trials (RCTs) on CVD risk factors (LDL-C and on clinical endpoints) focuses on the beneficial effects of specific macro- and micro-components of a plant-based diet (vegetable fats, DF, and PS) in the management of dyslipidemia and the role of plant-based dietary patterns in cardiovascular (CV) health and briefly discusses aspects of environmental sustainability. Dietary fatty acids, DF, and PS have been reviewed in detail because of the proven evidence for cholesterol-lowering and CVD risk benefits and because they are specifically referred to in dietary recommendations for CVD prevention [2]. Literature databases like MEDLINE were used as the main sources for searching relevant literature.

While health benefits of these individual dietary components have been addressed in specific reviews and meta-analyses, the information provided in this review addresses them in the context of a plant-based diet/dietary pattern and contributes to a better understanding of the increasingly important role of a plant-based diet in the prevention of CVD. In the following sections, specific components of a plant-based diet in the management of dyslipidemia and prevention of CVD are discussed in detail.

## 2. Dietary Fats

### 2.1. Dietary Sources and Fat Quality

There is general consensus that fat quality, i.e., the fatty acid composition of dietary fat, is more important than fat quantity (total amount of fat) in the management of dyslipidemia and the prevention of CVD. The fat quality of foods is determined by the content of different fatty acids. Fat from animal origin such as fatty meat, butter, full-fat dairy, as well as the tropical oils coconut and palm oil, are typically rich in saturated fatty acids (SAFA). Meat and dairy foods are the main food sources of SAFA in Western diets [11]. In contrast, plant-based fats, i.e., vegetable oils, are generally rich in unsaturated fatty acids. Unsaturated fatty acids can be monounsaturated (MUFA), such as oleic acid and polyunsaturated (PUFA). Plant-based sources of PUFA are predominately n-6 (omega-6) fatty acids such as linoleic acid and some n-3 (omega-3) fatty acids such as α-linoleic acid. Very long-chain n-3 fatty acids such as eicosapentaenoic (EPA) and docosahexaenoic acid (DHA) come from marine sources (fish, fish oil, and algae). Trans fatty acids (TFA), unsaturated fatty acids with double bonds in the trans configuration, are naturally found in butter, full-fat dairy, and meat from ruminants like beef, sheep, and goat. Other sources of TFA are partially hydrogenated vegetable oils. TFA are commonly found in commercial baked goods like pastries, convenience foods, and battered or deep-fried foods. TFA from partially hydrogenated oils have been removed from many foods because of their adverse effects on blood lipids and CVD risk [12], leading to a substantial decrease in TFA intake, which is typically now less than 1% of energy [13]. TFA and SAFA raise LDL-C but TFA also lowers HDL-C concentrations and therefore has the most unfavorable effects amongst dietary fatty acids [12].

Based on dietary intake data, SAFA consumption is still higher and PUFA consumption lower than recommended [11,14]. For CVD prevention, it is recommended that a decrease in SAFA intake should be accompanied by an increase in PUFA intake, but this is not always achieved at a population level [14].

### 2.2. The Role of Saturated, Monounsaturated, and Polyunsaturated Fatty Acids on Blood Lipids and CVD Risk

Replacing SAFA with unsaturated fatty acids in the diet lowers LDL-C without affecting HDL-C and TG. The LDL-C lowering effect is larger when SAFA is replaced by PUFA compared to MUFA [15,16,17]. Replacing SAFA with carbohydrates, i.e., consuming a low-fat, high carbohydrate diet, lowers both LDL-C and HDL-C and raises fasting TG, and therefore does not improve the overall blood lipid profile [15,17]. The most beneficial effect on blood lipids is therefore achieved by replacing SAFA with unsaturated fats. Supplemental intake of the very long-chain n-3 PUFA (EPA and DHA), from fish oil have no substantial effect on LDL-C but dose-dependently lower TG concentrations [13].

In view of effects on CVD risk and clinical endpoints, both observational, i.e. prospective cohort studies and RCTs with clinical endpoints, have shown that reducing SAFA intake lowers the risk of CVD and CHD events. There is clear evidence that partial replacement of SAFA with unsaturated fatty acids, especially vegetable oil PUFA (mainly n-6 linoleic acid and the plant-based n-3 fatty acid α-linolenic acid) lowers the risk of CVD, mainly the risk of CHD [13,17]. There is insufficient or limited evidence for effects on CVD or CHD risk when SAFA is replaced by MUFA, carbohydrates, or proteins, and when the replacement nutrient is not specified [13]. Replacing 5% of total energy intake (% TE) of SAFA with PUFA was found to lower the risk of CHD by about 10%, while evidence is inconclusive due to lack of adequate studies to quantify the CHD risk benefit of MUFA [13,17]. More recently, data from two large cohort studies found a significantly lower CHD risk when 5% TE intake from SAFA, TFA, and refined carbohydrates was replaced by MUFA from plant sources (vegetable oils, nuts, and seeds), while replacement with MUFA from animal sources (red and processed meats and dairy foods) was not associated with lower CHD risk [18]. In addition, data from two other cohort studies has shown that replacing 5 g/day of dietary fats like margarine, mayonnaise, butter or dairy fat with a higher intake of olive oil, which is rich in MUFA, was associated with a 5–7% lower risk of CHD and total CVD [19]. The Prevención con Dieta Mediterránea (PREDIMED) trial also studied the effect of consuming MUFA, in the form of olive oil, as part of a Mediterranean (MED)-type diet on CVD outcomes. The MED-type diet supplemented with either extra-virgin olive oil or with nuts significantly lowered major CVD events by around 30% compared to a low-fat diet [20]. It should, however, be noted that these dietary patterns differ in more aspects than just dietary fat and fatty acid intake.

Even though marine n-3 PUFA are not the focus of this review, observational studies have consistently shown that higher intakes of fish, fish oil, and EPA and DHA is associated with lower risk of CVD, especially CHD risk, in the general population [13,21]. While evidence from RCTs for the primary prevention of CVD is weak, higher intakes of EPA and DHA have been shown to lower CVD risk in RCTs in secondary prevention [13,21].

An analysis based on prospective cohort studies has further shown that replacing SAFA with PUFA, MUFA, or whole-grain (high-quality) carbohydrates was associated with a lower CHD risk, while replacing SAFA with refined carbohydrates and sugars (low-quality carbohydrates) was not associated with CHD risk [22]. Furthermore, a higher PUFA intake was associated with a lower CHD risk compared to higher intakes of refined carbohydrate and sugar. These data support that PUFA-rich plant-based fats as well as whole grain products as part of a predominantly plant-based diet are beneficial for CV health.

For management of dyslipidemia not all plant-based fats are beneficial, especially not those that are rich in SAFA such as coconut oil. Despite the widespread popularity of coconut oil and its alleged health benefits, its consumption significantly raises LDL-C compared with other vegetable oils as summarized in a recent meta-analysis [23]. Thus, coconut oil should not be recommended as a healthy vegetable oil for CVD risk reduction, despite its HDL-C-raising effect [23,24].

In addition to the beneficial effects on dyslipidemia, emerging data also show that unsaturated fatty acids, especially n-6 PUFA, favorably affect blood glucose and insulin resistance, therefore helping to reduce the risk of T2DM, a risk factor for CVD [13,17].

### 2.3. Recommendations for Dietary Fat Intake

Quality of dietary fat intake is key for the prevention of CVD more so than quantity. Recommendations for total fat intake typically range from 20 to 35% TE; intakes exceeding 35–40%TE should be avoided as they are usually associated with an increased intake of SAFA [25,26]. Reducing SAFA intake to <10% TE and replacing SAFA with unsaturated fatty acids (MUFA and PUFA) is the recommendation in most food-based dietary guidelines aiming to prevent CVD [2,27,28]. Some guidelines advise SFA intake <7% TE, especially in the presence of hypercholesterolemia (Table 1) [2]. TFA increases the risk of CVD [2,28,29], and intake should be avoided (Table 1). Intake of n-3 PUFA, especially EPA and DHA, is not part of dietary recommendations to lower TC and LDL-C concentrations. Nevertheless, some guidelines do recommend an intake of 200–500 mg/day of EPA and DHA as part of a heart healthy diet [21] or advise 1 to 2 portions of fatty fish per week [30]. For specific TG-lowering, high doses (2–4 g/day) of n-3 fatty acid supplements are recommended either alone or as an adjunct to other lipid-lowering therapy [2,21,30].

### 2.4. Food-Based Dietary Guidelines and How a Plant-Based Diet Can Improve the Quality of Dietary Fat Intake

Food-based dietary guidelines advise a more plant-based diet and recommend eating less foods of animal origin such as meat, butter, and full-fat milk and cheese [9]. They also advise switching to leaner meat and low-fat dairy products and replacing animal fats with vegetable oils and vegetable oil-based fats. As a result, pre-dominantly plant-based diets typically deliver less total fat, especially less SAFA and TFA, more unsaturated fats, and more plant-based proteins, DF, micronutrients (vitamins and minerals), and phytonutrients. Regular fish consumption, especially fatty fish, is also advised as a part of a plant-based, heart-healthy dietary pattern.

## 3. Dietary Fibers (DF)

### 3.1. Types of DF and Dietary Sources

DF consists of a wide range of plant-based compounds (carbohydrate polymers with ≤10 monomeric units) that vary in their physical and chemical properties. They have no nutritional value as such but play an important role in the regulation of different physiological functions in the body. DF are typically categorized into (water) soluble (SF) and insoluble fibers (IF) and are not hydrolyzed by digestive enzymes and hence not fully digested in the human gut [31]. IF include cellulose, hemi-celluloses, and lignin. They affect gut function by facilitating transit time of foods, normalizing bowel movements, increasing stool bulk, and preventing constipation. SF include pectin, beta-glucans, gums such as guar or konjac mannan, and mucilages like psyllium. They dissolve in water and form viscous gels in the gut lumen and so partially delay or reduce the absorption of carbohydrates, dietary fats, and cholesterol. IF are found mainly in vegetables, potatoes, nuts, and whole grain products such as wheat bran; sources of SF are vegetables, legumes, fruits such as apples, pears, citrus fruits, and cereals like oat and barley.

In addition to gut health, DF intake provides several health benefits and observational evidence has shown that a higher DF intake is associated with lower risk of CVD, T2DM, obesity, and certain forms of cancer [32,33].

Commonly, Western populations do not reach the recommended DF daily intakes. Grain-based foods contribute most to dietary DF intake with bread being by far the largest grain source followed by breakfast cereals. Vegetables, potatoes, and fruits also contribute substantially to DF intake [34].

In the context of this review, the beneficial effects of DF, and more specifically selected SF, on the risk of CVD and underlying risk factors such as dyslipidemia are detailed.

### 3.2. Effect of Specific DF on Blood Lipids and CVD Risk and Underlying Mechanism of Action

The first meta-analysis by Brown et al. [35] summarized the cholesterol-lowering effects of SF, i.e., oat products, psyllium, pectin, and guar gum. SF intake between 2–10 g/day was associated with modest but statistically significant decreases in TC and LDL-C, with no significant difference between the SF. Since then, several meta-analyses as discussed below have summarized the cholesterol-lowering effects of specific SF such as beta-glucan from oats and barley, psyllium, and glucomannan. A recent review found that increased DF intake significantly lowered TC and LDL-C, modestly but significantly decreased HDL-C, and had no effect on TG concentrations [36]. Further, there was no evidence that the type of DF, i.e., SF or IF or the way DF are administered, i.e., via supplements or foods, influenced the cholesterol-lowering effect [36].

Beta-glucan: Beta-glucan is a viscous SF found in oats, barley, edible mushrooms, and (baker’s) yeast. A recent meta-analysis found that a median intake of 3.5 g/day of oat beta-glucan modestly lowered LDL-C by −0.19 mmol-L (95% CI: −0.23 to −0.14) or 4.2% and non-HDL-C by −0.20 mmol/L (95% CI: −0.26 to −0.15) or 4.8% compared with control diets [37]. An earlier meta-analysis found an LDL-C reduction of −0.25 mmol/L or 6% with a median daily intake of 5.1 g beta-glucan from oats [38].

Barley beta-glucan was also shown to lower blood cholesterol. A median intake of 6.5 and 6.9 g/day of barley beta-glucan, respectively, lowered LDL-C by −0.25 mmol/L (95% CI: −0.30 to −0.20) or 7% and non-HDL-C by −0.31 mmol/L (95% CI: −0.39 to −0.23) or 7% compared with control diets [39]. Similar effects were reported in a previous meta-analysis [39]. There was no clear dose–response relationship for barely beta-glucan [39], while a significant inverse association between dose and LDL-C lowering was found for oat-beta-glucan [37]. The established LDL-C lowering effect of beta-glucan, especially from oats, has resulted in approval of health claims for oat beta-glucan and its LDL-C lowering effect or CVD risk reduction benefit in Europe, USA, Canada, and Australia/New Zealand referring to an intake of at least 3 g/day of oat beta-glucan.

Psyllium: Psyllium is a viscous SF from the husk of the *Plantago ovata* seed and is a common fiber supplement. Two recent meta-analyses [40,41] found that daily intake of 10.2 g psyllium significantly lowered LDL-C by −0.28 mmol/L (95% CI: −0.21 to −0.31) [39] and -0.33 mmol/L (95% CI: −0.38 to −0.27) [40]; non-HDL-C was found to be lowered by −0.39 mmol/L (95% CI: −0.50 to −0.27) [41]. No clear dose–response relationship was observed, suggesting that the cholesterol-lowering benefit of psyllium intakes of ≥10 g/day will not result in bigger LDL-C lowering [41]. The recommended intake of psyllium for an optimal cholesterol-lowering and heart health benefit is 7 g/day SF from 10.2 g psyllium husk, based on the approved US FDA health claim.

Glucomannan: Glucomannan, also known as konjac mannan, is one of the most viscous DF. Its main source is the tuberous root of the konjac plant. Typically, glucomannan is consumed in the form of capsules next to some food formats like bars or biscuits. A recent meta-analysis found that daily intake of ~3 g glucomannan significantly lowered LDL-C by −0.35 mmol/L (95% CI: −0.46 to −0.25) or 10% and non-HDL-C by −0.32 mmol/L (95% CI: −0.46 to −0.19) or 7% [42]. There was no indication of a dose–response effect with glucomannan intakes between 2.0–15.1 g/day. An intake of 4 g/day glucomannan is the minimal dose of an approved health claim in Europe for maintaining a healthy blood cholesterol concentration.

Summarizing, an intake of 4–10 g/day of different types of SF is required to achieve a 5–10% reduction in LDL-C without substantially affecting HDL-C and TG concentrations.

SF with high viscosity and water-binding capacity form viscous gels in the intestinal lumen which decrease absorption of macronutrients as well as of cholesterol and bile acids and subsequent increased fecal excretion [32]. An impaired reabsorption and increased excretion of bile acids stimulates bile acids synthesis in the liver which consequently lowers circulating cholesterol concentrations in the blood. Furthermore, colonic fermentation of SF by gut bacteria produces short-chain fatty acids, and increased concentrations of circulating propionate may contribute to cholesterol lowering by decreasing cholesterol synthesis in the liver [32,43].

In view of the effects on CVD risk and clinical endpoints, observational studies have consistently shown that higher intakes of DF and dietary patterns high in DF are associated with a reduced risk of CVD as well as T2DM and obesity, also risk factors for CVD [32]. Meta-analyses of prospective cohort studies have shown a significant association between DF intake and lower risk of all-cause mortality [44] and mortality from CVD and CHD [43,44]. An additional DF intake of 7–10 g/day was inversely linked to a reduction in CVD mortality by 9% and CHD by 9–11% [44,45]. A recent umbrella review of systematic reviews and meta-analyses of observational studies concluded that there is convincing evidence that higher DF intake is associated with a lower risk of CVD, and particularly of coronary artery disease (CAD) and CVD-related death [33].

DF intake from cereals was significantly associated with lower CVD risk [43,44], while results for other fiber sources seem less conclusive. Kim and Je found that DF intake from legumes also showed an inverse association with CVD risk, while DF intake from vegetables and fruits failed to show an association [44]. Conversely, Threapleton et al. report an inverse association of vegetable fiber intake and CVD risk [44]. In particular, IF intake seems to be linked with lower risk of CVD while such a benefit was not convincingly seen for SF intake [44,45]. A recent prospective French cohort study supports that a higher intake of DF is associated with a decrease in CVD incidence and mortality. Both SFs and IFs were associated with lower risk of T2DM, while SFs were also associated with lower CVD risk. Amongst different DF sources, DFs from fruits were inversely associated with CVD risk [46]. Noteworthy, there are no RCTs on the effects of specific DF such as beta-glucan that have been shown that lowering LDL-C affects CVD outcomes.

### 3.3. Food-Based Dietary Guideline Recommendations for DF Intake

Recommendations for DF intake typically refer to total fiber intake. A healthy diet should contain more than 25 g/day of DF and most European countries recommend 25 and 30 g/day [34]. In the EFSA scientific opinion on dietary reference values, a DF intake of 25 g/day would be adequate for normal laxation in adults while intakes higher than 25 g/day would be necessary to reduce risk of CHD, T2DM, and to improve weight maintenance [34].

Usually, no recommendations are made for intakes of specific fiber types such as SF. Nevertheless, in view of approved health claims for maintaining or lowering blood cholesterol concentrations, daily intakes of 3 g beta-glucan from oats, oat bran, barley, or barley bran, 6 g pectin, 10 g guar gum, and 4 g glucomannan could be recommended. The 2019 ESC/EAS guidelines for the management of dyslipidemia recommend a DF intake of 25–40 g/day, including ≥7–13 g of SF, preferably from wholegrain products, e.g., oats and barley (Table 1) [2].

Adopting a predominantly plant-based diet helps to achieve the daily recommended intake of DF; in particular, vegan and vegetarian-type diets are rich in DF from various plant-based sources such as whole grains and seeds, legumes, vegetables and fruits, and nuts.

## 4. Phytosterols

### 4.1. Dietary Sources

Phytosterols (PS), comprising plant sterols and stanols, are compounds similar in structure and function to cholesterol. Principal PS are sitosterol, campesterol, and stigmasterol and their saturated counterparts sitostanol and campestanol. They occur naturally in all plant-based foods and are found in vegetable oils (especially unrefined oils), vegetable oil-based margarines, seeds, nuts, cereal grains, legumes, vegetables and fruits next to various foods and food supplements with added PS [47]. Daily intakes of PS with habitual diets typically range between 200 and 400 mg [48]. Consuming diets that emphasise plant-based foods results in higher PS intakes. With vegetarian- or vegan-type diets, PS intake can increase up to 600 mg/day [49]. With a MED-type diet such as the PREDIMED diet [20] or the dietary approaches to stop hypertension (DASH) diet [50], PS intakes of 500–550 mg/day can be achieved. Higher PS intakes such as a recommended intake of 2 g/day for LDL-C lowering [2] can only be achieved by consuming food products enriched with PS such as fat-based spreads and margarines, dairy-type foods like milk, yogurt, and yogurt drinks, or food supplements with added PS. A PS intake of about 2 g/day can be realized with the Portfolio diet through the consumption of a daily serving of a PS-added margarine next to consuming SF, soy, and other vegetable proteins and nuts, i.e., almonds [51].

### 4.2. Cholesterol-Lowering Efficacy and Underlying Mechanisms of Action

The TC and especially LDL-C lowering properties of PS in humans were discovered in the early 1950s [52]. Several meta-analyses have summarized their LDL-C lowering efficacy based on numerous RCTs [53,54,55,56]. The most recent meta-analysis including 124 clinical studies (with 201 study arms) concluded that PS intake significantly lowers LDL-C in a dose-dependent manner by 6–12% with intakes of 0.6–3.3 g/day without affecting HDL-C. [56]. PS intakes exceeding 3 g/day have only little additional benefit as the LDL-C lowering effect is expected to taper off because the underlying mechanism of action—inhibition of cholesterol absorption—is a saturable process. Nevertheless, RCTs with PS intakes greater than 4 g/day are limited; thus, it remains speculative whether the dose-response relationship would continue with higher PS intakes [56]. PS intake was also found to lower atherogenic apo-lipoproteins (apo) such as apo-B and apo-E and to increase anti-atherogenic apo-lipoproteins like apo-AI and apo-CII as summarized in a recent meta-analysis [57].

PS are effective in both healthy and diseased individuals and their LDL-C lowering benefit has been demonstrated in adults and children with familial hypercholesterolemia, in patients with T2DM, and individuals with the metabolic syndrome [58]. Furthermore, PS are shown to be effective in various types of food formats such as fat-based foods like spreads and margarines, dairy-type foods, and food supplements including capsules and tablets, thereby offering a variety of choices to achieve the recommended daily PS intake for a cholesterol-lowering benefit [47]. Intake occasion and frequency are critical factors for an optimal LDL-C lowering efficacy. Thus, PS should be consumed with a (main) meal and twice daily [47].

Meta-analyses have also found a significant TG-lowering effect whereby the effect was more pronounced in individuals with higher baseline TG concentrations [59]. Hence, PS offer a dual blood lipid benefit especially in individuals with dyslipidemia such as patients with T2DM or the metabolic syndrome [59].

Partial inhibition of intestinal absorption of (dietary and biliary) cholesterol is the key mechanism for the cholesterol-lowering effect of PS, with several underlying mechanisms including displacing cholesterol from mixed micelles due to limited capacity to embody sterols, interfering with transport-mediated processes of sterol uptake and stimulating cholesterol excretion via the transintestinal excretion [60]. An intake of 2 g/day of PS reduces cholesterol absorption by 30–40%, resulting in a subsequent 10% lowering of circulating LDL-C [61].

PS intake can also be a useful adjunct to lipid-lowering medication. Statins inhibit hepatic cholesterol synthesis and PS inhibit intestinal cholesterol absorption; thus, combining PS and statins leads to an additive LDL-C lowering effect [61,62]. Additional effects on LDL-C lowering have also been reported when combining PS with fibrates or with ezetimibe [61,63].

Although there are no RCTs showing the effects of long-term PS intake on CVD outcomes, e.g., CV events; it seems reasonable that PS intake may lower CVD risk based on the established LDL-C lowering effect.

### 4.3. Recommendations for PS Intake

Based on the proven plasma LDL-C lowering effect and the absence of adverse effects, consumption of 2 g/day PS as an adjunct to a healthy diet is one of the recommended dietary interventions for the management of dyslipidemia (Table 1) [2,64,65]. Foods with added PS may be considered i) for individuals with high serum cholesterol at intermediate or low global CVD risk who do not (yet) qualify for drug treatment, ii) as adjunct to drug (statin) therapy, in high- to very high-risk patients who fail to achieve LDL-C target goals or could not be treated with statins, and iii) in adults and children (>6 years) with familial hypercholesterolaemia, in line with current guidelines [2,61].

## 5. Combinations of Natural Lipid-Lowering Compounds as Part of a Heart-Healthy Diet

Combining PS (2 g/day) with different types of SF, e.g., 3 g/day oat beta-glucan has been shown to lead to additional LDL-C lowering when combined in a single food [66]. Further, a combination of 10 g/day psyllium and 2.6 g/day PS added to cookies leads to a substantial reduction in LDL-C [67].

In addition, the combination of 3.3 g/day PS with a healthy diet (low in total and saturated fat) shows an additive effect of PS to that of the diet alone [68]. By combining PS with other plant-based, cholesterol-lowering compounds or foods, further reductions in LDL-C can be achieved as observed with the Portfolio diet. This plant-based dietary pattern combines (per 2000 kcal energy intake) 20 g/day of viscous DF from oats, barley, psyllium, eggplant, okra, apples, oranges, or berries, about 50 g/day of plant protein from soy products or pulses (beans, peas, chickpeas, and lentils), 42 g/day of nuts (tree nuts such as almonds and peanuts), and 2 g/day PS in the form of a PS-enriched margarine [51,69]. Under controlled settings, an LDL-C lowering effect of 30% could be achieved with the Portfolio diet, an effect comparable to that achievable with a low-dose statin [51]. Based on a meta-analysis, the Portfolio dietary pattern significantly lowers LDL-C by -0.73 mmol/L (95% CI: -0.89 to -0.56) or by ~17%. Non-HDL-C, apo B, TC, TG, as well as systolic and diastolic blood pressure, were also significantly lowered [69].

A dual blood lipid lowering benefit of lowering both LDL-C and TG concentrations was achieved with the intake of 2 g/day PS and a minimum of 1 g/day EPA/DHA from fish oil [70].

## 6. The Impact of Plant-Based Dietary Patterns on CV and Planetary Health 

### 6.1. Dietary Patterns and CV Health

Historically, dietary recommendations for CVD prevention focused on single nutrients like lowering SAFA or increasing DF intake, or on specific foods like eating more fish, whole grains, nuts, fruits, and vegetables, and less meat. Today, the focus is on the role of dietary patterns for the management of dyslipidemia and lowering CVD risk [71,72]. A healthy dietary pattern is typically described as a predominantly plant-based diet. Observational study evidence supports the beneficial effect of single plant-based foods as well as fatty fish, and the adverse effects of animal-based foods, on CVD risk [73,74]. Since foods are typically consumed in combinations, it is more reasonable to look at health benefits of a complete dietary pattern.

Several dietary patterns have been found to lower CVD outcomes and risk factors such as blood lipids, i.e., TC and LDL-C or blood pressure. These include traditional diets prevailing in the MED and in Nordic countries [75,76,77,78,79], dietary patterns intended to control CVD risk factors like the DASH diet [50] and Portfolio diet [51] as well as vegetarian- or vegan-type dietary patterns. A common characteristic of these dietary patterns is that they emphasize plant-based foods with reduced animal food consumption.

The MED dietary pattern refers to the traditional diet of Greece, Crete, and Southern Italy with (virgin) olive oil as the main dietary fat source as a key characteristic [72,75,78]. Further characteristics are summarized in Table 2. The Nordic diet emphasizes the intake of locally grown vegetables like cabbage and potatoes, whole grains and cereals such as oats, rye, and barley, locally grown seasonal fruits such as berries, next to rapeseed oil and fatty fish (Table 2) [72,77,78]. The DASH diet emphasizes amongst others higher intakes of vegetables, fruits and fat-free or low-fat dairy foods (Table 2) [50,79]. The Portfolio diet is a plant-based diet that emphasises the intake of four known cholesterol-lowering foods, i.e., viscous DF, plant proteins from soy and legumes, nuts, and PS (Table 2) [51,69]. The healthy vegetarian dietary pattern is based on a variety of vegetables, legumes, soy products, fruits, and whole grains, occasionally dairy foods, eggs, and fish, but no meat and poultry. A strict vegan dietary pattern excludes all animal-based foods (Table 2) [72,73,80].

### 6.2. Effect on Blood Lipids

Both observational studies and RCTs have found that dietary patterns emphasizing consumption of plant-based foods have beneficial effects on blood lipids, especially on TC and LDL-C.

Next to lowering systolic and diastolic blood pressure, for which the DASH diet was originally designed, the DASH diet was also found to lower TC and LDL-C. An umbrella review of systematic reviews and meta-analyses concluded that TC was lowered by −0.20 mmol/L (95% CI: −0.31, −0.10) and LDL-C by −0.10 mmol/L (95% CI: −0.20 to −0.01) without affecting HDL-C and TG [79,81]. The observed cholesterol-lowering benefit of the DASH diet may be attributable to the high intake of DF from the consumption of fruits, nuts, legumes, and whole grains, and the lower intake of saturated fat.

The Portfolio diet combining four recognized cholesterol-lowering foods/food components with a background diet low in total fat (≤30% TE) and saturated fat (<7% TE) and low in dietary cholesterol (<200 mg/day) led to clinically relevant benefits in lowering cholesterol and other CVD risk factors. A meta-analysis of RCTs has shown that TC was lowered by −0.81 mmol/L (95% CI: −0.98 to −0.64) or 12% and LDL-C by -0.73 mmol/L (95% CI: -0.89 to -0.56) or 17% [69]. Non-HDL-C was lowered by -0.83 mmol/L (95% CI: −1.03 to −0.64) or 14% and TG by −0.28 mmol/L (95% CI: −0.42 to −0.14 mmol/L) or 16%; all compared to a low total fat, low saturated fat, and low cholesterol diet. The LDL-C lowering effect of the Portfolio diet was found to be 21% in efficacy trials and 12% in effectiveness trials. Other CVD risk factors like systolic and diastolic blood pressure and C-reactive protein were also lowered [69].

Following a MED or a Nordic dietary pattern is also associated with beneficial blood lipid effects. Based on a meta-analysis of RCTs, the Nordic diet lowered TC by −0.39 mmol/L (95% CI: −0.76 to −0.01) and LDL-C by −0.30 mmol/L (95% CI: −0.54 to −0.06) with no significant changes in HDL-C and TG [82,83]. Beneficial effects on reducing systolic and diastolic blood pressure were also seen [83]. The beneficial effects of the Nordic dietary pattern can be attributed to the high DF intake and the low intake of saturated fat. Likewise, the MED dietary pattern was found to significantly lower LDL-C by −0.07 mmol/L (95% CI: −0.13 to−0.01) and TG by −0.46 mmol/L (95%CI: −0.72 to −0.21) [83]. Small or no reductions in TC and/or LDL-C were reported in a recent review that compared a MED diet intervention vs. either no intervention or another dietary intervention in primary prevention of CVD [84]. Blood pressure is also modestly reduced by the MED diet, but effects are less than those observed with the Nordic diet [83].

Several meta-analyses addressed the effects of a vegetarian dietary pattern on blood lipids. Two meta-analyses of RCTs found that vegetarian diets significantly lowered TC by −0.32 to −0.36 mmol/L and LDL-C by −0.32 to −0.34 mmol/L [85,86]. HDL-C was also significantly lowered by −0.09 to −0.10 mmol/L while TG were not significantly altered [85,86]. Another recent meta-analysis of RCTs studying vegetarian diet patterns and lipid risk factors in T2DM found a reduction in LDL-C of −0.12 mmol/L (95% CI: −0.20 to 0.04) with no significant effects on HDL-C and TG [83]. Taken together, vegetarian diet patterns, particularly vegan diets, are associated with lower blood cholesterol concentrations. That vegetarian diets are low in total and especially saturated fat, low in dietary cholesterol, and particularly high in DF intake may explain their cholesterol-lowering effect. The higher intake of phytochemicals such as PS, phenolics compounds, and carotenoids with vegetarian/vegan diet patterns may further contribute to this effect.

### 6.3. Effects on CVD Risk and Outcomes

Evidence for beneficial effects of dietary patterns on CVD risk and related outcomes derives mostly from observational studies; RCTs that studied the effect of dietary patterns on CVD-related endpoints are rare or non-existent.

There is no direct evidence from observational studies for a CVD risk benefit, e.g., on CVD incidence or mortality of the Portfolio diet. Nevertheless, the combined effects of that dietary pattern on CVD risk factors such as LDL-C and blood pressure as observed in RCTs was assumed to decrease the estimated 10-year CHD risk by ~13% [69].

The DASH diet that is well-accepted for its blood pressure lowering effect was found to reduce CVD incidence by 20%, CHD incidence by 21%, and stroke incidence by 19% [79,83]. These CVD risk-related benefits are attributable to the substantial reductions in blood pressure and in other CVD risk factors, i.e., TC and LDL-C.

The effects of the MED diet on CVD risk have been assessed in several meta-analyses of observational studies. Rosato et al. [87] found in their meta-analysis of prospective studies a 19% lower risk for unspecified CVD, a 30% lower risk for CHD/acute myocardial infarction, a 27% lower risk for unspecified stroke, and 18% lower risk for ischemic stroke when comparing the highest vs. the lowest adherence to this dietary pattern based on a MED diet score [72,87]. Another meta-analysis of prospective studies reports a risk reduction (RR) in total CVD, CHD, and stroke mortality of 0.79 (95% CI: 0.77, 0.82), 0.83 (95% CI: 0.75, 0.92), and 0.87 (95% CI: 0.80, 0.96), respectively, next to a lower CHD incidence (RR: 0.73; 95% CI: 0.62, 0.86) and stroke incidence (RR: 0.80; 95% CI: 0.71, 0.90), comparing the highest vs. lowest categories of MED diet adherence [88]. A meta-analysis of three RCTs revealed that the MED diet is associated with a 38% lower risk of total CVD and a 35% lower risk of total myocardial infarction (MI) but a nonsignificant reduction in CVD mortality [83,88]. Especially the PREDIMED trial, a primary prevention RCT that investigated a modified MED diet supplemented with either extra virgin olive oil or nuts compared to a reduced-fat control diet, revealed an approximately 30% lower incidence of a composite of CVD endpoints (MI, stroke, and death from CV causes) with these MED diet patterns [20]. A re-analysis of the original outcome data assessing individual endpoints revealed a reduction in stroke incidence but little or no effect on total and CVD mortality or MI [20,84]. In their review, Rees et al. [84] concluded that there is still some uncertainty regarding the effects of a MED-style diet on clinical endpoints and CVD risk factors for both primary and secondary prevention.

Less, though more conflicting, evidence for CVD risk-related effects are reported for the Nordic diet. Adhering to a healthy Nordic diet was associated with a lower risk of CVD in men and women from the Danish Diet, Cancer and Health cohort [89,90] but not in women of the Swedish Women’s Lifestyle and Health cohort [91]. A recent systematic review and meta-analysis of prospective studies assessing the Nordic diet reports a modest reduction in CVD risk (RR: 0.93; 95% CI, 0.88, 0.99) and stroke risk (RR: 0.87 95% CI, 0.77, 0.97) but not in CVD mortality [83].

Meta-analyses of observational study evidence support that a vegetarian diet pattern is associated with a 22% lower CHD mortality while no association was found for CVD or stroke mortality. Furthermore, vegetarian diets are associated with a 28% reduced risk of CHD [80,83]. These results are comparable to a previous meta-analysis reporting a 25% risk reduction in CHD incidence and mortality but no significant effects for CVD and stroke and all-cause mortality [92].

## 7. Dietary Patterns and Environmental Aspects

Dietary choices clearly contribute to the risk for developing CVD. Evidence supports the intake of specific nutrients, food groups, or certain dietary patterns to positively influence dyslipidemia and to promote the prevention of CVD. Next to their impact on CV health, dietary patterns also impact the environment in various ways. Plant-based dietary patterns have been shown to have a smaller impact on climate change (greenhouse-gas emissions), fresh-water use, cropland use (deforestation), and biodiversity loss [8,93] than consumption of animal-based foods such as (red and processed) meat. A recent global modeling study combined analyses of nutrient level, diet-related, and weight-related chronic disease mortality, and environmental impact in different sets of diet scenarios for more than 150 countries [94]. Four different energy-balanced diets were found to reduce environmental impact, nutrient deficiencies, and diet-related mortality. All four dietary patterns were predominately plant-based diets with limited red and processed meat intake. These patterns were described as flexitarian, pescatarian, vegetarian, and vegan diets [94].

It must be noted that not all plant-based diets are necessarily beneficial in view of diet-related chronic diseases such as CVD risk [74]. Observational evidence from cohort studies found that plant-based diets containing higher amounts of refined grains, juices/sugar-sweetened beverages, sweets, potatoes/fries were associated with an increased CHD risk, while plant-based diets including higher amounts of whole grains, fruits, vegetables, legumes, nuts, and vegetable oils were associated with lower risk of CHD [74,95]. While predominately plant-based diets have beneficial effects on CV risk, their impact on body weight and prevention or treatment of obesity are less clear [78]. Hence, consuming an energy-balanced diet in view of avoiding weight gain and obesity seems crucial.

There are several possibilities for adopting a predominately plant-based dietary pattern based on personal food taste and preference as well as individual needs. Characteristics for choosing a predominantly plant-based healthy diet are summarized in Table 3.

## 8. Current Recommendations for a Healthy Dietary Pattern

The CV health benefits of certain dietary patterns have been recognized by numerous dietary and clinical practice guidelines. For instance, the DASH dietary pattern is recognized by the 2015–2020 dietary guidelines for Americans [96], the AHA/ACC guideline on lifestyle management to reduce cardiovascular risk [27], the Canadian Cardiovascular Society guidelines for the management of dyslipidemia [64], and by the ESC/EAS 2019 guidelines for the management of dyslipidaemias [2]. The Canadian Cardiovascular Society [64] also mentions the Portfolio diet as a healthy dietary pattern. Adopting a healthy MED diet pattern for the promotion of healthy eating and the prevention of CVD is acknowledged by the 2015–2020 dietary guidelines for Americans [96], the 2019 ACC/AHA guideline on the primary prevention of CVD [27], and by the ESC/EAS 2019 guidelines for the management of dyslipidaemias [2]. Eating a plant-based Nordic diet as a healthy alternative to the MED-dietary pattern is advocated by the Nordic nutrition recommendations [97]. A healthy vegetarian eating pattern is mentioned by the 2015–2020 dietary guidelines for Americans [96] and the Canadian Cardiovascular Society guidelines for the management of dyslipidemia [64].

Notably, the emphasis of eating a predominantly plant-based diet as a healthy eating pattern to prevent diet-related chronic diseases such as CVD is a common theme of many food-based dietary guidelines [9,96]. Another driving factor for recommending a more plant-based diet relates to environmental sustainability [8,9]. Nevertheless, the environmental aspects of dietary patterns are not yet widely addressed in food-based dietary guidelines, only a few countries have so far included environmental sustainability in their guidelines [78].

## 9. Conclusions

This review describes the beneficial effects of specific macro- and micro-components of a plant-based diet (vegetable fats, DF, and PS) in the management of dyslipidemia and CVD prevention. Moreover, the role of common plant-based dietary patterns in CV health is discussed.

The protective effect of predominately plant-based dietary patterns such as the MED and Nordic diet or the DASH and Portfolio diet on CVD risk and related risk factors, e.g., LDL-C, is associated with specific plant-based foods which are known for their CV health benefits [72,74,75,76,79]. These foods include fruits, vegetables, legumes, whole grains, nuts, and seeds [73,74]. Plant-based foods are typically rich in unsaturated fatty acids, DF, plant proteins and various micronutrients like vitamins and phytonutrients such as PS and polyphenols; they are low in saturated fats and often low in energy density compared to foods from animal sources [74]. The beneficial effects of these plant-based food components are explained by different underlying mechanisms which influence the development of CVD either directly or indirectly by influencing risk factors such as dyslipidaemia. For instance, replacing saturated fats in the diet with unsaturated fatty acids like MUFA and PUFA is known to lower LDL-C [13,16]. Observational studies and RCTs with clinical endpoints have further shown that replacing SAFA with especially vegetable oil PUFA lowers the risk of CVD, mainly the risk of CHD [13,17] whereas evidence for effects on CVD or CHD risk when SAFA is replaced by MUFA, carbohydrates, or proteins is insufficient or limited [13]. DF, especially viscous SF like beta-glucan, reduce the intestinal absorption of cholesterol and re-absorption of bile acids and produce short-chain fatty acids in the colon which may affect hepatic cholesterol synthesis, all contributing to a LDL-C-lowering effect [43]. Observational studies have shown a significant association between DF intake and lower risk of all-cause mortality [44] and mortality from CVD and CHD [43,44]. PS also inhibit intestinal cholesterol absorption resulting in lower circulating concentrations of LDL-C [61]. Although there are no RCTs showing the effects of long-term intake of specific DF like beta-glucan or PS on CVD outcomes, e.g., CV events, it seems reasonable that their intake may lower CVD risk based on the established LDL-C lowering effect. Replacing animal protein with plant protein has also been shown to lower LDL-C [98].

Noteworthy, not all plant-based dietary patterns are equally effective in lowering CVD risk as not all plant-based foods have beneficial CV effects [74,95]. Hence, the quality of plant-based foods and food components plays an important role. For instance, a dietary pattern with more refined grains than whole grains or more sugars-sweetened foods and beverages have been linked with a higher CVD risk. The same dietary patterns for which a positive impact on dyslipidemia and the prevention of CVD has been shown can also help reduce the impact of food choices on environmental degradation [8]. Hence, shifting to a more plant-based dietary pattern will not only improve CV health but will also be more environmentally sustainable. While plant-based, e.g., vegetarian, dietary patterns are generally perceived positively because of their benefits on health (CVD prevention) and the environment, there are also several barriers that hinder the switch to, and maintenance of a plant-based diet. Common barriers include health concerns that a plant-based diet may lack specific nutrients, reluctance to change dietary behavior, and enjoyment of eating meat and animal-based foods [99,100].

A limitation of this narrative review is that only selected macro- and micro components of a plant-based diet are described while other components such as vitamins and other phytonutrients like polyphenols with proposed CV benefits were not considered. Nevertheless, dietary fatty acids, DF, and PS were chosen because of their proven evidence for LDL-C-lowering and CVD risk benefits and because they are specifically referred to in dietary recommendations for CVD prevention [2]. While health benefits of these individual dietary components have been addressed before, this review addresses them in the context of a plant-based dietary pattern. As mostly observational study evidence is available on CVD risk and clinical endpoints, more evidence from RCTs demonstrating such benefits for these dietary components in the context of a plant-based diet would be desirable, despite the challenges of carrying out long-term, controlled dietary intervention studies.

## Figures and Tables

**Table 1:** Impact of specific dietary changes in the management of dyslipidemia with focus on lowering low-density lipoprotein cholesterol (LDL-C) concentration.

Dietary Component	Specific Recommendation	Magnitude of Effect ^1^	Strength (Level) of Evidence ^2^
Dietary fat			
Reduce intake of saturated fatty acids (SAFA)	<10% of total energy intake (TE)<7% TE in the presence of hypercholesterolemia	++	***
Exchange SAFA with unsaturated fatty acids	Lower SAFA and increase intake of mono- (MUFA) and polyunsaturated fatty acids (PUFA)	++	***
Avoid intake of dietary trans fat	Reduce to <1%TE	++	***
Reduce intake of dietary cholesterol	<300 mg/day	+	**
Dietary fiber (DF)			
Increase total DF intake	25–40 g/day	++	***
Increase intake of soluble fibers, e.g., beta-glucan	≥7–13 g/day as part of total DF intake	++	***
Phytosterols	≥2 g/day	++	***

Modified based on the 2019 ESC/EAS guidelines for the management of dyslipidaemias [2]. ^1,2^ Magnitude of effect and strength of evidence refer to the impact of each dietary change on lowering TC and LDL-C concentrations. ^1^ Magnitude of effect: ++ = 5–10% reduction; + = <5% reduction. ^2^ Level of evidence: *** Data derived from multiple randomized clinical trials (RCTs) or meta-analyses; ** Data derived from a single RCT trial or from large non-randomized studies.

**Table 2:** Healthy dietary patterns for the management of dyslipidemia and the prevention of cardiovascular diseases (CVD).

Healthy Dietary Pattern	Background/Definition	Key Characteristics
Mediterranean (MED) diet	Traditionally based on dietary patterns typical of Crete, Greece, and Southern Italy in the early 1960s. No uniform definition of a MED diet, but MED dietary patterns emphasize plant-based foods and olive oil as main dietary fat source. Modified versions were studied in the PREDIMED trial ^1^.	Eating plenty of fruits, vegetables, legumes, (whole) grains, nuts; olive oils as main oil for daily use; moderate intake of fish; poultry and dairy foods like yogurt and cheese; eating less red meat, meat products and sweets; allows wine (in moderation) with meals. The MED diet is high in dietary fat and especially monounsaturated fatty acids but low in saturated fat.
Nordic diet	A dietary pattern comparable to the MED diet that emphasises traditional, locally grown, and seasonal foods of the Nordic countries. Developed as a diet to address health concerns such as obesity and taking local food culture, environmental aspects, and sustainability into account ^2^.	Emphasizes locally grown, seasonal foods; eating plenty of fruits, e.g., berries, vegetables, e.g., cabbage, legumes, potatoes, whole grains, e.g., oats and rye breads, nuts, seeds, fish and seafood, low-fat dairy, rapeseed oil, and, in moderation, game meats, free-range eggs, cheese, and yogurt; rarely eating red meats and animal fats; avoiding sugar-sweetened beverages, added sugars, processed meats. The Nordic diet is especially rich in dietary fiber and low in sugar and sodium.
Dietary approaches to stop hypertension (DASH) diet	A prescribed dietary pattern originally developed to lower blood pressure as studied in the DASH clinical trials ^3^.	Eating plenty of fruits, vegetables, legumes, whole grains; including fat-free or low-fat dairy products, fish, poultry, nuts, seeds, and vegetable oils; limiting fatty meats, tropical oils, sweets, sugar-sweetened beverages. The DASH diet is low in saturated fat, dietary cholesterol, salt (sodium), and high in dietary fiber, potassium, and calcium.
Portfolio diet	A predominately plant-based, vegan-type diet developed to further include a portfolio of foods/food components that are known to lower total and LDL-cholesterol ^4^.	Eating a diet low in fat (≤30% of energy), especially saturated fat (<7% of energy), and high in fruits and vegetables with the addition of four plant-based, cholesterol-lowering foods: 50 g/day plant protein from various soy foods, legumes like beans, chickpeas, lentils; 45 g/day (about a handful) nuts such as peanuts, almonds; 20 g/day viscous soluble fiber from oats, barley, eggplant, okra, apples, berries, oranges, and psyllium; 2 g/day plant sterols from enriched foods such as spreads, dairy-type foods, or from supplements.
Vegetarian/vegan diet pattern	Dietary patterns of specific population groups that were adapted based on observational studies and randomized controlled trials.	Eating plenty of fruits, vegetables, legumes, whole grains, nuts and seeds, specific foods, e.g., soy products and excluding meat and poultry and partly also dairy foods, eggs, and fish; lacto/ovo-vegetarians eat eggs and dairy products; lacto-vegetarians consume dairy products, ovo-vegetarians eat eggs, and pesco-vegetarians eat fish and seafood; vegans completely refrain of all animal-based foods including meat, poultry, eggs, dairy foods, and fish. Vegetarian/vegan diets are high in dietary fiber, and typically low in total and saturated fat, intake of n-3 fatty, acids, iron, and vitamin B_12_.

Adapted in parts from Hemler and Hu, 2019 [74], Zampelas and Magriplis, 2019 [72], Magkos et al. 2020 [78]. ^1^ Estruch et al. [20], ^2^ Bere and Brug [76], ^3^ Appel et al. [50], ^4^ Jenkins et al. [51].

**Table 3:** Characteristics of a predominantly plant-based healthy diet for management of dyslipidemia and CVD prevention.

Foods and Food Group-Related Recommendations	
**Eat more**	
Vegetables	Eat plenty of vegetables * including a wide variety from all colors
Legumes	Choose from a variety of legumes and pulses
Fruits	Eat plenty of (fresh or frozen) fruits * including berries; limit intake of fruit juices
Nuts and seeds	Choose from a variety of unsalted tree nuts and peanuts
Whole grain	Eat a variety of whole grains such as whole grain bread, pasta, brown rice
**Eat adequately/moderately**	
Healthy fats	Limit saturated fats like butter, full-fat cheese, and cream; avoid trans fats; choose vegetable oils and fats (margarine) rich in unsaturated fatty acids
Milk and dairy foods	Choose no- or low-fat over full-fat milk, cheese, and other dairy foods
Fish and seafood	Choose sustainable varieties of fish and seafood
Poultry and eggs	Eat occasionally and choose products from welfare-oriented animal husbandry
**Limit eating**	
Meat	Eat less red and processed meats
Sweets and sugar-sweetened beverages	Limit intake of sweets and sugar-sweetened beverages
**Nutrient-specific recommendations**	
Reduce saturated fats by replacing them with mono- and polyunsaturated fats	SAFA should be replaced with MUFA or PUFA to reduce LDL-C.
Increase total dietary fiber (DF) intakeand esp. intake of soluble DF	Consume 25-45 g of DF per day of which 5–15 g of soluble fibers from foods rich in these fibers for an LDL-C lowering effect
Limit salt intake	Limit salt intake of less than 5 g/day
**Specific advice to further lower LDL-C**	
Consider phytosterols as an adjunct to a healthy diet	Consume about 2 g/day phytosterols through foods/foods supplements with added phytosterols
Increase intake of soluble fiber such as beta-glucan from oat and barley	Consume about 3 g/day beta-glucan from foods like oat bran, oatmeal, barley flakes, pearl barley

Complied based on dietary recommendations described in Mach et al. [2], the UK Eat Well guide (https://www.gov.uk/government/publications/the-eatwell-guide), the 2015–2020 dietary guidelines for Americans [96]. * Plenty refers to 5 portions of fruits and vegetables per day.
